# Localized ultrasonic stimulation using a piezoelectric micromachined ultrasound transducer array for selective neural differentiation of magnetic cell-based robots

**DOI:** 10.1038/s41378-025-00900-y

**Published:** 2025-03-20

**Authors:** Seonhyoung Kim, Dong-in Kim, Hong Goo Yeo, Gyudong Lee, Jin-young Kim, Hongsoo Choi

**Affiliations:** 1 Department of Robotics & Mechatronics Engineering, Daegu Gyeongbuk Institute Science and Technology (DGIST), Daegu, 42988 Republic of Korea; 2 Department of Advanced Materials Engineering, Sun Moon University, Asan-si, 31460 Republic of Korea; 3 Division of Nanotechnology, Daegu Gyeongbuk Institute Science and Technology (DGIST), Daegu, Republic of Korea; 4 Division of Biotechnology, Daegu Gyeongbuk Institute Science and Technology (DGIST), Daegu, Republic of Korea; 5 DGIST-ETH Microrobotics Research Center, Daegu, 42988 Republic of Korea

**Keywords:** Electrical and electronic engineering, Electronic devices

## Abstract

Targeted stem cell delivery utilizing a magnetic actuation system is an emerging technology in stem cell engineering that efficiently targets stem cells in specific areas in vitro. However, integrating precise magnetic control systems with selective neural differentiation has not yet been widely considered for building successful neural networks. Challenges arise in creating targeted functional neuronal networks, largely due to difficulties in simultaneously controlling the positions of stem cells and selectively stimulating their differentiation. These challenges often result in suboptimal differentiation rates and abnormalities in transplanted neural stem cells. In contrast, ultrasound stimulation has superior tissue penetration and focusing capability, and represents a promising noninvasive neural stimulation technique capable of modulating neural activity and promoting selective differentiation into neuronal stem cells. In this study, we introduce a method for targeted neural differentiation using localized ultrasonic stimulation with a piezoelectric micromachined ultrasound transducer (pMUT) array. Differentiation was assessed quantitatively by monitoring neurite outgrowth as the ultrasound intensity was increased. The neurite length of cells ultrasonically stimulated for 40 min was found to have increased, compared to the non-stimulated group (119.9 ± 34.3 μm vs. 63.2 ± 17.3 μm, respectively). Targeted differentiation was confirmed by measuring neurite lengths, where selective ultrasound stimulation induced differentiation in cells that were precisely delivered via an electromagnetic system. Magnetic cell-based robots reaching the area of localized ultrasound stimulation were confirmed to have enhanced differentiation. This research demonstrated the potential of the combination of precise stem cell delivery with selective neural differentiation to establish functional neural networks.

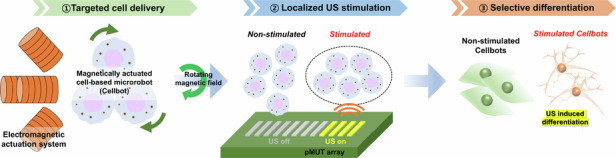

## Introduction

Neurodegenerative diseases such as Parkinson’s disease and Alzheimer’s disease are caused by irreversible damage to neurons in the nervous system, resulting in movement disorders or memory impairment^1–3^. Due to the limited regenerative capability of mature neural cells, the human brain struggles to self-repair after nerve injury. Neuronal stem cells have the potential for regeneration and functional recovery by renewing and differentiating into neuronal cells at sites of damage^4–6^. Stem cell treatment has been performed to regenerate and recover the nervous system^7,8^. Conventional surgical transplantation of stem cells is associated with risks. In addition, the transport of stem cells as therapeutic agents has low delivery efficiency and difficulty in selectively delivering to the target region^9,10^. A number of methods and delivery systems have been developed for the transport and transplantation of stem cells into target lesions^11–14^. Recently, the use of a magnetically actuated delivery system has emerged as a promising approach to enable the precise positioning of cells at specific sites^11,15^. In addition, compared to actuation systems using other external energy sources, the ability of magnetic fields to penetrate the human body harmlessly has been exploited to successfully deliver stem cells to the central nervous system^10,13^. However, the low differentiation efficiency of transplanted and transported stem cells into functional neuronal cells poses challenges for their use in therapeutic applications^16,17^. Therefore, methods to enhance the differentiation efficiency of stem cells are required to achieve the full therapeutic potential of stem cell therapy.

Among the available strategies to promote neuronal cell differentiation, neural cell stimulation is being explored using various electrical, mechanical, and acoustic energy sources^18–22^. Electrical stimulation was shown to affect neurite outgrowth and guide neurite orientation but requires the surgical implantation of electrodes into the brain^19,23^. In contrast, noninvasive interventions using electrical fields induced by magnetoelectricity or piezoelectricity have low spatial resolution and can result in energy transfer to off-targeted sites^18,24^. Mechanical stimulation approaches involving the activation of mechanosensitive pathways through contact and vibrational forces have also been reported. For example, piconewton-scale forces generated using an atomic force microscope (AFM) can induce differentiation with the induction of a neuronal response via ion channel gating^20^. However, it is unlikely that direct contact methods using mechanical stimuli can be adapted to improve target cell differentiation. Physical stimulation using repeated vibrational motion induced by a magnetic field was shown to be an efficient method for enhancing neuronal differentiation in the deep tissue of the brain^25,26^. Unlike light and ultrasound, this method disperses the magnetic field in three-dimensions, making it difficult to localize stimuli to the target area.

Given these limitations, ultrasound is a suitable energy source for targeted brain stimulation due to its ability to penetrate deeply into tissues and its ability to focus with good spatial resolution^27,28^. Moreover, the established safety of ultrasound stimulation has enabled biomedical applications of diagnostic imaging and deep brain stimulation, further highlighting its potential for precise and safe modulation of neuronal activity^29–31^. However, commercial ultrasound transducers used in previous studies are often constrained by size and lack the flexibility to adjust the physical distance from the target area to target neuronal cells.

To address these challenges, in this study, we developed a method for targeted cell delivery and selective differentiation by combining an electromagnetic actuation system and a selective ultrasound simulation system using a miniaturized piezoelectric micromachined ultrasound transducer (pMUT) array. The pMUT array allows localized stimulation and reduces the size constraints for stimulation through the application of a small, flexible array design. Using a pMUT array, we utilized a miniaturized stimulator, distinct from those mainly employed in neuromodulation, to localize the effects of ultrasound on selective cell differentiation^32,33^. The effects of ultrasound on cell differentiation were validated by assessing neurite outgrowth as a function of the applied ultrasound dose, given that neurite length can be used to indicate the characteristics of differentiation^34^. As a proof-of-concept of localized ultrasound stimulation for stem cell differentiation, in vitro experiments were conducted to verify the feasibility of selective differentiation at the target site after magnetic delivery of neural stem cells by an electromagnetic system. Superparamagnetic iron oxide nanoparticle (SPION) cluster-loaded SH-SY5Y cells were successfully delivered to the target region via external magnetic fields. Targeted differentiation by ultrasound was assessed by measuring neurite length using immunostaining in the ultrasound-stimulated area. Finally, we demonstrated the potential of integrating magnetic stem cell delivery with selective ultrasound stimulation using a pMUT array.

## Results and discussion

### Fabrication of the pMUT array

The geometry of the pMUT array was designed to confirm the effect of ultrasound stimulation on cell differentiation while considering the biological experimental environment. The pMUT array had dimensions of 1 mm × 7 mm, enabling the observation of both stimulated and non-stimulated areas within the cell culture imaging dish (observation window diameter: 21 mm) while maintaining uniformity across the array. The membrane size and spacing between pMUT elements were designed to achieve high-fill factor arrays by minimizing pitch size with high frequency under the constraints of the fabrication process.

**Fig. 1:**
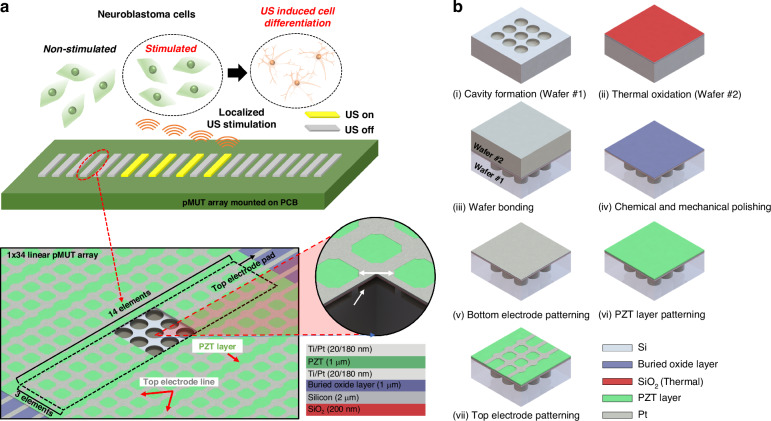
Schematic representation of the design and fabrication of the one-dimensional (1D) piezoelectric micromachined ultrasound transducer (pMUT) array for localized ultrasound stimulation. **a** Schematic image of the 1D pMUT array. **b** Fabrication of the pMUT array via a wafer bonding process

Top and cross-sectional views of the fabricated pMUT array for localized ultrasound stimulation are shown in Fig. 1a. The proposed one-dimensional (1D) pMUT array consisted of 34 channels with 42 pMUT circular membrane elements 60 μm in diameter in each channel. The distance between each channel and pMUT elements was 20 μm, which was designed for the application of ultrasound stimulation to a localized area. The ultrasound was generated by activating each channel to stimulate cells in the desired region. Following the wafer bonding process, the pMUT was composed of a Pb(Zr,Ti)O_3_ (PZT) layer 1-μm thick sandwiched between 20 nm titanium (Ti) and 180-nm platinum (Pt) electrodes on the structural layer with a prepared cavity wafer (wafer #1). The geometric parameters of pMUT array are listed in Table 1.

**Table 1:** Geometric parameters of pMUT array

Center frequency (*f*_*0*_) in air	9.22 MHz
Total array size	1 mm × 7 mm
No. of channels	34
No. of elements per channel	42
Membrane diameter	60 μm
Element-to-element distance	10 μm
Channel-to-channel distance	10 μm
Pitch size	210 μm

The overall fabrication process flow is illustrated in Fig. 1b. The fabrication process began with the preparation of two different wafers, a cavity wafer and SiO_2_− grown silicon on an insulator (SOI) wafer with a 2 μm device layer and a 1 μm buried oxide (BOX) layer. First, the cavity with a depth of ~10 μm was defined on the bare silicon wafer using the deep reactive-ion etching (DRIE) process (step i). The SiO_2_ layer was thermally grown on the SOI wafer (wafer #2) for the Si–SiO_2_ interface with reliable bonding strength. Then, the cavity wafer was prebonded to the SOI wafer in a vacuum at room temperature (step ii). The bonded wafer pair was annealed at 1050 °C for 60 min in a furnace to enhance the bonding strength using a commercial wafer bonding setup from the National NanoFab Center (Daejeon, Republic of Korea). The Si handling layer of the SOI wafer was ground to ~20 μm by chemical mechanical polishing and XeF_2_ isotropic etching to minimize physical damage to the membrane (step iv).

A stack consisting of a bottom electrode of Ti/Pt (20 nm/180 nm), a piezoelectric PZT layer (1 μm), and a top electrode of Ti/Pt (20 nm/180 nm) was deposited using a sputtering system (SRN-100; Sorona, Daegu, Republic of Korea) and then patterned via lift-off on the BOX layer after fully removing the residual Si layer (steps v–vii). A ferroelectric PZT is usually used in pMUT devices as the piezoelectric active layer due to its high piezoelectric properties^35,36^. The PZT layer was grown on the Pt bottom electrode by sputtering using a Pb(Zr_0.48_,Ti_0.52_)O_3_ ceramic target (Kojundo Chemical Laboratory, Saitama, Japan). The resultant PZT film (~500 nm thick) was annealed at 550 °C and 650 °C for 30 min in a muffle furnace (KVD206; KSM Component, Gimpo, Republic of Korea). The PZT layer deposition step was repeated twice to achieve a PZT layer 1 μm thick. Finally, a bilayer of Ti/Pt was sputtered and patterned on the PZT layer as the top electrode. The pMUT was used for cell stimulation after applying a coating of Parylene C (3 μm thick) for electrical insulation and water permeability.

### Characterization of pMUT

**Fig. 2:**
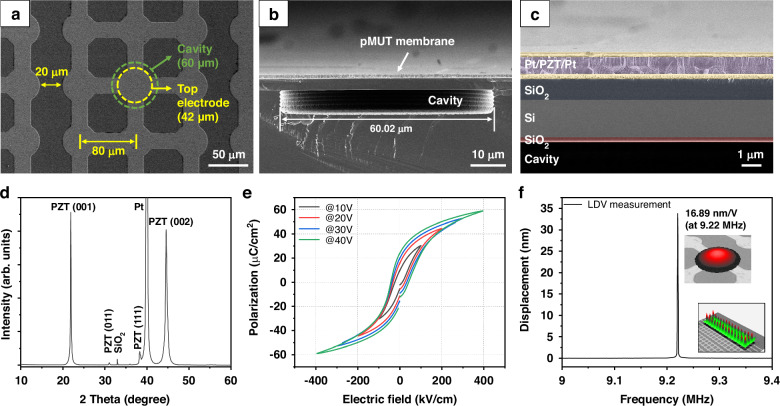
Characterization of the fabricated pMUT. **a** Top-view scanning electron microscopy (SEM) image of the fabricated pMUT array. **b** Cross-sectional SEM image of a pMUT element. **c** Magnified cross-sectional SEM image of the structural layer of a pMUT element. **d** X-ray diffraction (XRD) pattern of a Pb(Zr,Ti)O_3_ (PZT) film deposited on the bottom electrode. **e** Polarization–electric field curve of the deposited PZT film. **f** Frequency response of the pMUT membranes measured by scanning laser-Doppler vibrometer (SLDV) with a 2 *V*_pp_ sinusoidal signal at the first resonant frequency (9.22 MHz). Inset: Measured deflection profile of a single pMUT element and array with a 2 *V*_pp_ sinusoidal signal applied at the first resonant frequency

The fabricated 1D pMUT array was composed of 34 parallel channels. Scanning electron microscopy (SEM) images showing top and cross-sectional views of the fabricated pMUT array are presented in Fig. 2a–c. A circular top electrode 42 μm in diameter was patterned on the structural layer with a circular cavity 60 μm in diameter. The pitch of the pMUT elements was 80 μm, and the channel-to-channel distance was 20 μm. The cross-sectional SEM image in Fig. 2b shows that the DRIE process successfully formed the cavity. In a cross-sectional view, the Pt/PZT/Pt stack of the pMUT membrane was confirmed to have been deposited successfully on the SiO_2_/Si/SiO_2_ structural layer without significant cracks.

X-ray diffraction (XRD) measurement was conducted to evaluate the crystallinity of the PZT layer (Fig. 2d). The PZT thin film crystallized in the ferroelectric perovskite structure with a preferred orientation of (100) without any secondary phases. The hysteresis curves in Fig. 2e show the typical shape of a ferroelectric PZT layer with a polycrystalline perovskite structure. The measured coercive fields obtained using an applied voltage of 40 V were 41.1 and −42.7 kV/cm, respectively. The remnant polarization was 22.97 μC/cm^2^. After characterization of the ferroelectric properties of the PZT layer, the vibrational properties of pMUT were evaluated by laser-Doppler vibrometer (LDV) in air. The displacement sensitivity of the fabricated pMUT was measured by applying a sinusoidal wave of 2 *V*_pp_ at the first resonance frequency (Fig. 2f). The displacement sensitivity in the center of the membrane was 16.89 nm/V at 9.22 MHz. The measured resonance frequency of pMUT was within the sub-MHz frequency range, which has been used to activate mechanosensitive ion channels and influence the differentiation of neuronal stem cells^37^. The vibrational topography showed that the pMUT elements in the channel were uniformly operated.

### Acoustic properties of the pMUT

**Fig. 3:**
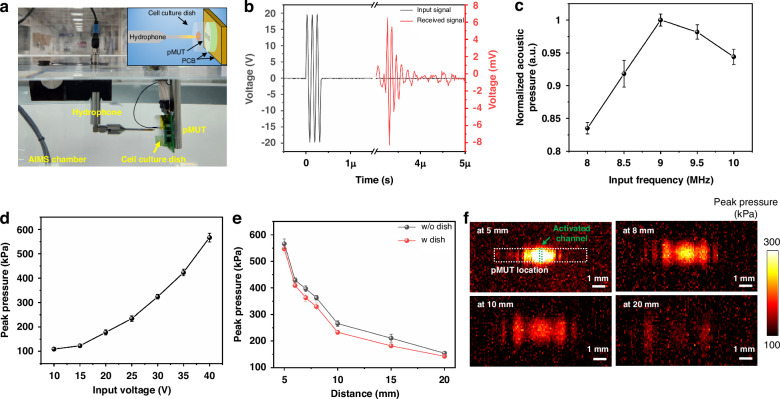
Acoustic characterization of the fabricated pMUT. **a** Photograph of the acoustic experimental setup with the acoustic intensity measurement system (AIMS). Inset: Schematic image of experimental setup. **b** Time response of the acoustic signal from one activated channel at the center position in the pMUT array with 40-*V*_pp_ at 9 MHz. **c** Peak pressure as a function of the input frequency to pMUT (*n* = 5). **d** Peak pressure as a function of the input voltage to pMUT at 9 MHz (*n* = 5). **e** Peak pressure according to horizontal distance from the hydrophone and cell culture dish (40 *V*_pp_, 9 MHz, *n* = 5). **f** Acoustic intensity distribution with different horizontal distances from the hydrophone by the pMUT driven at 40 *V*_pp_ and 9 MHz

The acoustic pressure of the fabricated pMUT was measured in water using an acoustic intensity measurement system (AIMS) (AIMS III; Onda Corp., Sunnyvale, CA, USA) at room temperature. Figure 3a shows the experimental setup for acoustic characterization of pMUT, including the hydrophone and pMUT array, which were aligned horizontally. The acoustic pressure measured from the hydrophone (HNP-0400; Onda Corp.) is shown in Fig. 3b. One of the 34 channels at the center of the pMUT array was activated using three cycles with various input voltages and frequencies by a function generator. The acoustic pressure as a function of input frequency, ranging from 8 MHz to 10 MHz, demonstrated that the resonant frequency with the peak pressure in water was around 9 MHz (Fig. 3c). At the resonant frequency, the peak pressure as a function of driven voltage to pMUT was calculated from the signal received from the hydrophone (Fig. 3d). The peak pressure increased from ~108 –~566 kPa at 5 mm as the driving voltage was increased from 10-to 40-*V*_pp_. The acoustic characteristics were evaluated according to the horizontal distance from the hydrophone (5 –20 mm) with and without a cell culture dish consisting of a 180 μm thick polymer membrane for cell stimulation. The acoustic intensity decreased markedly from ~566 –~153 kPa with increasing distance due to attenuation in water. However, there was no significant difference in acoustic intensity measured with or without a cell culture dish, indicating that the effect of the cell culture dish on the acoustic intensity was negligible. The measured acoustic pressure field distributions in the x–y plane are shown as a function of the distance from the hydrophone in Fig. 3f. When one channel at the center of pMUT was activated, the measured maximum and minimum acoustic pressures at 5 mm were 0.53 and 0.22 MPa, respectively, within the boundary of the activated pMUT channel. The distance used for cell stimulation was 1 mm, which allowed stronger ultrasound stimulation than the measured acoustic intensity at 5 mm, which was the minimum measurable distance with our system. The measured sound pressure above the activated channel of the pMUT decreased markedly with increasing horizontal distance from the hydrophone. At a horizontal distance of 1 mm from the center position of the activated channel, the acoustic intensity was reduced by half of the maximum value. These results showed that ultrasound generated by the pMUT could be used for cell stimulation in localized regions.

### Effects of ultrasound stimulation using the pMUT array on cell differentiation in SH-SY5Y cells

**Fig. 4:**
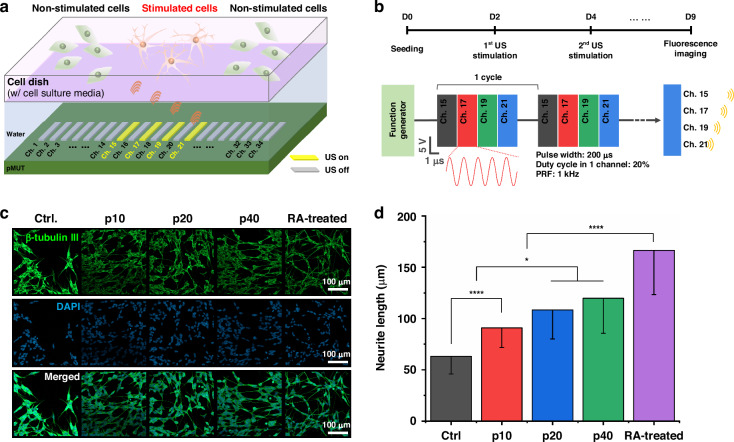
Evaluation of the effect of ultrasound stimulation using the pMUT on cell differentiation. **a** In vitro experimental setup for ultrasound stimulation of SH-SY5Y cells using the pMUT. **b** Experimental timeline and schematic of the electrical signal applied to the pMUT for ultrasound stimulation. **c** Immunofluorescence images of SH-SY5Y cells labeled with anti-β-III tubulin antibody (green) and stained with *4*’*,6-diamidino-2-phenylindole* (DAPI, blue) in non-stimulated (Ctrl.), ultrasound stimulated (p10, p20, and p40), and retinoic acid (RA) treatment groups. **d** Mean neurite lengths of SH-SY5Y cells determined from immunofluorescence images (*n* = 3 dishes in all comparisons). *P < 0.5; ****P < 0.0001

The effects of ultrasound stimulation on cell differentiation were confirmed using SH-SY5Y cells. As shown in Fig. 4a, ultrasound stimulation was performed by placing SH-SY5Y cells on a dish above the pMUT array, and the selected pMUT channels (15, 17, 19, and 21) corresponding to the targeted stimulation region were activated. Considering the width of a single pMUT channel and the horizontal width of the transmitted ultrasound beam, each channel was selected and operated with the appropriate spacing to minimize the overlap of the ultrasound-stimulated areas. Sequential pulsed ultrasound was performed to avoid creating an area of excessive irradiation when the channels were operated simultaneously. Sequential pulsed ultrasound for cell stimulation was generated by applying 15-*V*_pp_ at 9 MHz with a pulsed repeated frequency (PRF) of 1 kHz (Fig. 4b). The ultrasound exposure dose was varied by using different exposure times of 10, 20, and 40 min (p10, p20, and p40, respectively) to evaluate dose effects on cell differentiation. Stimulation was conducted twice, on days 2 and 4, after cell seeding. On day 5, after the second ultrasound stimulation, fluorescence imaging of SH-SY5Y cells was performed to analyze neurite length and morphology (Fig. 4c). To characterize neuronal differentiation, neurite outgrowth of the stimulated cells was compared with that of non-stimulated cells (Ctrl.) and chemically differentiated cells using differentiation medium containing 10 μM retinoic acid (RA) as a control group. In immunofluorescence imaging, the biomarker β-III tubulin was used to confirm the differentiation of neurons^38,39^. The ultrasound stimulation group (p10, p20, and p40) exhibited significant neurite elongation in comparison with the non-stimulated group. To confirm the effect of ultrasound stimulation on cell differentiation, the mean neurite length was measured on randomly captured fluorescent images within the stimulated region in all dishes^40^. The results showed significantly enhanced neurite extension in p10, p20, and p40 to 90.9 ± 19.2, 108.5 ± 28.4, and 119.9 ± 34.3 μm, respectively, compared to the non-stimulated group (63.2 ± 17.3 μm) (Fig. 4d). Neurite length increased gradually with exposure time. The neurite length in the RA treatment group was 166.5 ± 43.0 μm, which was longer than in cells exposed to ultrasound stimulation for 40 min. However, neurite length in the experimental group would likely be optimized by further exploration of various ultrasound stimulation conditions.

### Fabrication of magnetic Cellbots

**Fig. 5:**
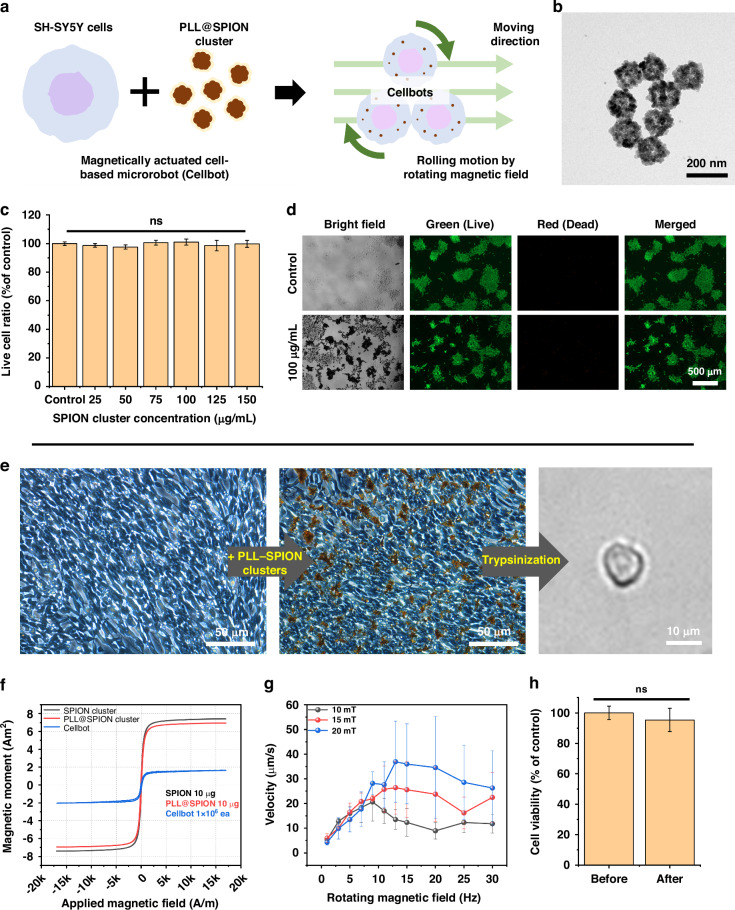
Fabrication and characterization of Cellbots. **a** Schematic of the fabrication of Cellbots and mechanism for Cellbot manipulation using a rotating magnetic field. **b** Transmission electron microscopy (TEM) image of superparamagnetic iron oxide nanoparticle (SPION) clusters after poly-l-lysine (PLL) coating. **c** Cytotoxicity analysis of PLL-SPION clusters toward SH-SY5Y cells at different concentrations (ns, not significant; *P* > 0.05; *n* = 6). **d** Fluorescence images of live/dead stained SH-SY5Y cells at different concentrations of PLL-SPION clusters. **e** Optical images of SH-SY5Y cells during the Cellbot fabrication procedure. **f** Magnetic hysteresis curves of 10-μg SPION clusters, 10-μg PLL-SPION clusters, and 1 × 10^6^ Cellbots. **g** Velocities of the Cellbots (*n* = 3). **h** Cell viability of Cellbots before and after magnetic manipulation

To verify our hypothesis regarding targeted delivery and selective differentiation, we investigated whether cell differentiation could be induced by ultrasound after delivery to the target site via an electromagnetic system. As shown in Fig. 5a, magnetically actuated cell-based robots (SH-SY5Y cells with poly-l-lysine [PLL]-coated SPIONs), referred to as “Cellbots,” were fabricated by cocultivation of SH-SY5Y cells and PLL-functionalized SPION (PLL-SPION) clusters. PLL is a polycation widely used to enhance the cellular uptake of nanoparticles^13,41^. After functionalization using PLL, the morphology of the PLL-SPION clusters was examined by transmission electron microscopy (TEM) (Fig. 5b). The results confirmed that the PLL-SPION clusters exhibited no significant changes in size or shape after functionalization, as reported previously^42^. The biocompatibility of the PLL-SPION clusters was examined through a cell viability assay and live/dead fluorescence imaging. SH-SY5Y cells were incubated in cell culture medium with PLL-SPION clusters at concentrations from 0 –150 μg/mL. The results showed no significant reduction in the live/dead cell ratio under the tested conditions (Fig. 5c). There was no significant difference in live/dead staining results between the control and PLL-SPION cluster groups (Fig. 5d).

To fabricate Cellbots, SH-SY5Y cells were incubated in a cell culture medium with 100 μg/mL PLL-SPION clusters for 4 h (Fig. 5e). Although loading large numbers of nanoparticles into the cells is advantageous for magnetic field manipulation, the aggregation of nanoparticles at high concentrations could be detrimental to Cellbot purification. Therefore, 100 μg/mL was considered an appropriate concentration and was used in Cellbot fabrication. After 24 h of cocultivation, SH-SY5Y cells transfected with magnetic nanoparticles were trypsinized after removing excessive aggregated unbound nanoparticles. The size of a single Cellbot was around 10 μm, and groups of Cellbots were delivered to the target region under the control of an external magnetic field. The magnetic properties of the Cellbot were confirmed by magnetic hysteresis measurements of the bare SPION clusters, PLL-SPION clusters, and Cellbots (Fig. 5f). For the PLL-SPION clusters, a reduction in the magnetic moment was observed due to the mass of the non-magnetic materials by the PLL polymer. The magnetic hysteresis of Cellbots exhibited a shape typical of the superparamagnetic properties of SPION clusters. From the measured data on PLL-SPION clusters and Cellbots, the quantity of nanoparticles absorbed by a single Cellbot was calculated as 2.94 pg.

The spherical Cellbots were directed to the target region through a rolling motion on the surface by applying a rotating magnetic field with an electromagnetic system. The velocity of a single Cellbot was assessed at various frequencies of the rotating magnetic field with strengths ranging from 10 –20 mT. The velocity increased linearly with frequency until it reached a critical point known as the step-out frequency. At a magnetic field strength of 20 mT, a single Cellbot achieved a peak velocity of 36.9 µm/s at 13 Hz and declined gradually thereafter. Variations in cell size and the nonuniform uptake of magnetic particles caused variability in the velocity measurements. However, a clear correlation was observed between the Cellbot velocity and the applied magnetic field, along with a decrease in velocity above the step-out frequency. The potential for cell damage caused by the applied magnetic field during magnetic manipulation was assessed by cell viability testing of the Cellbots. A magnetic field of 20 mT at 3 Hz was applied for 30 min to Cellbots on cell culture plates, and no significant difference in cell viability was observed compared to the control group (Cellbots without magnetic manipulation). Therefore, the effects of the magnetic field and magnetic manipulation on cell viability were considered to be negligible.

### Effects of ultrasound stimulation using the pMUT array on the differentiation of SH-SY5Y cells from Cellbots

**Fig. 6:**
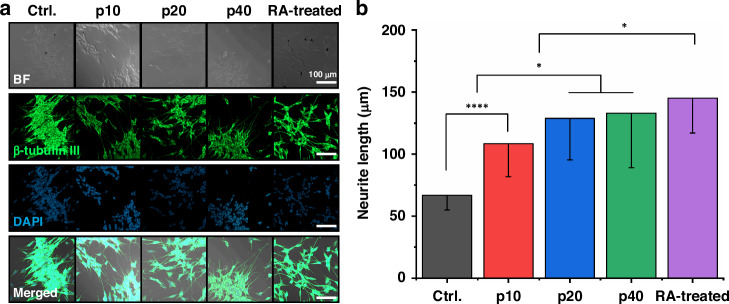
Evaluation of the effect of ultrasound stimulation using the pMUT array with SH-SY5Y Cellbots. **a** Immunofluorescence images of Cellbots labeled with anti-β-III tubulin antibody (green) and stained with DAPI (blue) in non-stimulated (Ctrl.), ultrasound stimulated (p10, p20, and p40), and retinoic acid (RA) treatment groups. **b** Mean neurite lengths of Cellbots determined from immunofluorescence images (*n* = 3 dishes in all comparisons). *P < 0.5; ****P < 0.0001

The effects of ultrasound stimulation on cell differentiation without SPION were discussed above. We conducted the same experiment using Cellbots, including SPION clusters (Fig. 6a), but without magnetic manipulation. The Cellbots were seeded and subjected to ultrasound stimulation under the same experimental conditions as outlined in Fig. 4b. Pulsed ultrasound stimulation of varying durations from 10 to 40 min was applied on days 2 and 4 after seeding. Immunofluorescence analysis comparing non-stimulated (Ctrl.) and ultrasound-stimulated groups (p40) showed that ultrasound stimulation enhanced cell differentiation, with a marked increase in neurite length in the experimental group with stimulation for 40 min (Fig. 6b). The results showed that interactions between nanoparticles and ultrasound, such as thermal effects or the generation of reactive oxygen species, did not significantly affect cell viability or differentiation^43,44^.

### Targeted differentiation of magnetically manipulated Cellbots in vitro

**Fig. 7:**
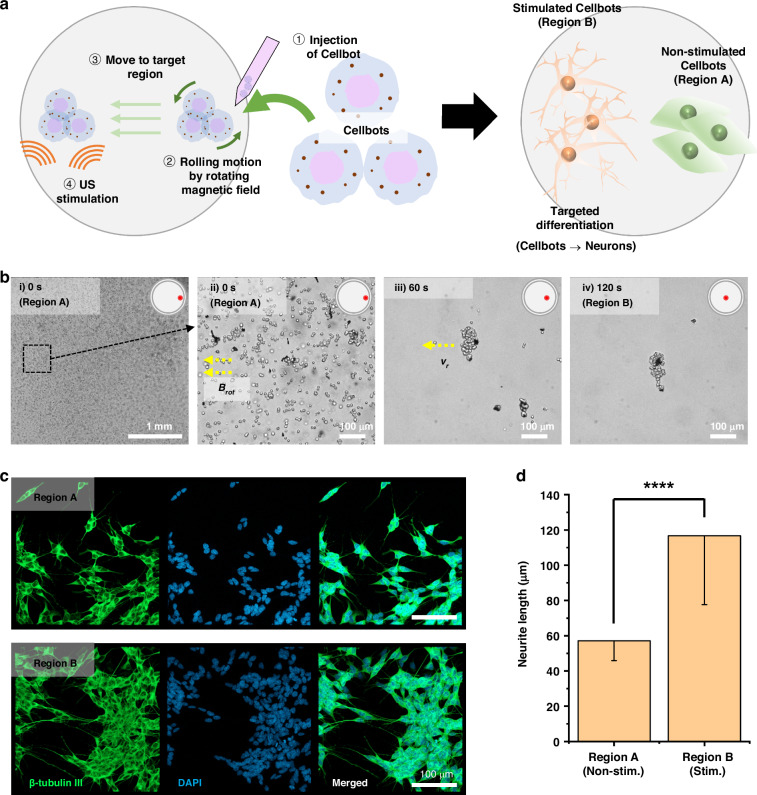
In vitro magnetic manipulation and selective differentiation of Cellbots by ultrasound stimulation at the target region. **a** Schematic view of targeted differentiation of Cellbots using ultrasound stimulation after magnetic manipulation. **b** Bright-field images of Cellbots migrating via a rolling motion from left to center under a rotating magnetic field. **c** Immunofluorescence images of Cellbots labeled with anti-β-III tubulin antibody (green) and stained with DAPI (blue) in the non-stimulated area (region A) and stimulated area (region B). **d** Mean neurite lengths of Cellbots determined on immunofluorescence images at non-stimulated and stimulated areas (*n* = 3 dishes in all comparisons). ****P < 0.0001

Cellbots with SPIONs were delivered to target regions using an electromagnetic actuation system, and cell differentiation was selectively promoted by ultrasound stimulation at the site of interest using a pMUT array aligned with the position of the delivered Cellbots. The experimental procedure for the in vitro manipulation of Cellbots toward a target region with selective ultrasound stimulation is illustrated in Fig. 7a. Initially, Cellbots were placed at the edge of the cell culture dish (region A). Following magnetic manipulation to the area of interest (region B), the effects of ultrasound stimulation on differentiation were evaluated by activating the pMUT on Cellbots that had reached their destination. As shown in Fig. 7b, the Cellbots were directed from region A to region B using a rotating magnetic field at 10 Hz with a field intensity of 20 mT. Cellbots were found either in small groups or isolated cells but clustered as they moved in the direction driven by the magnetic field (Fig. 7b-ii–iv). The Cellbots were then stimulated with ultrasound after a 2 day incubation period. In region B, Cellbots were ultrasonically stimulated for 40 min, which was found to be the most effective duration for enhanced differentiation. These Cellbots were then compared with non-stimulated Cellbots from region A after 9 days by confocal microscopy (Fig. 7c). The neurites in region B were significantly longer than those in region A (116.7 ± 39.2 µm vs. 57.2 ± 11.3 µm, respectively, *P* < 0.0001) (Fig. 7d). These observations confirmed that differentiation could be selectively induced by localized ultrasound stimulation of the target area. Furthermore, these findings suggested that combining ultrasound stimulation with a magnetic manipulation system could be applied to establish selective neural connections by delivering therapeutic stem cell agents to regions requiring neural connectivity and locally promoting differentiation with adequate ultrasonic energy at the target site.

## Conclusions

Targeted stem cell delivery with a magnetic actuation system has the potential for the reconstruction of neural networks and functional recovery at the region of interest. However, such systems have not been combined with the selective differentiation of stem cells following delivery to the target region to regenerate neuronal connections. In this study, we successfully demonstrated a strategy for targeted neural differentiation after targeted cell delivery using magnetic cell-based robots incorporating pMUT-based localized ultrasound stimulation for selective differentiation in the target region. The pMUT array consisting of 60 μm-sized elements was suitable for use as a miniaturized stimulator and allowed ultrasound stimulation in a localized area with sufficient ultrasonic energy to promote cell differentiation. The effects of ultrasound stimulation using the human neuroblastoma SH-SY5Y cell line indicated effective promotion of neurite outgrowth and neuronal differentiation, with the extent of differentiation increasing as the ultrasound dose increased. We further explored the integration of Cellbots magnetically manipulated with the pMUT array to achieve targeted neuronal differentiation. Cellbots with PLL-functionalized SPION clusters showed excellent biocompatibility and magnetic properties, enabling precise magnetic actuation to target regions using an electromagnetic system. Cellbots delivered to the target region were ultrasonically stimulated and showed enhanced neurite outgrowth compared to non-stimulated cells. This study demonstrated the potential for establishing selective neural connections by promoting differentiation in specific regions using ultrasound after magnetic Cellbot manipulation. The magnetic manipulation and ultrasound stimulation system could significantly advance targeted therapy in the treatment of neurological disorders and enhance neural engineering.

## Materials and Methods

### SEM, XRD, and P-E hysteresis loop measurement of pMUT

To confirm the structure of the fabricated pMUT, top- and cross-sectional views were obtained by FE-SEM (SU8230; Hitachi, Tokyo, Japan). The specimen for cross-sectional SEM imaging was mechanically prepared by cutting the pMUT array using a sharp diamond tip. Cross-sectional images were acquired from specimens that were clearly cut through the center of the membrane. XRD was conducted to confirm the crystallinity and phase of the PZT layer. The ferroelectric hysteresis loops (polarization vs. electric field) were characterized using a ferroelectric tester (Precision LC ii; Radiant Technologies, Albuquerque, NM, USA) with a range of electric field strengths (−400 to +400 kV/cm) at 1 Hz. The standard bipolar waveform was applied to the sample during hysteresis loop measurement.

### Vibrational characterization of the pMUT

The resonance frequency and displacement of the fabricated pMUT were measured using a scanning laser-Doppler vibrometer (SLDV) (MSA-500; Polytec GmbH, Waldbronn, Germany). The resonance frequencies of pMUT elements were evaluated by applying 2 *V*_pp_ periodic chirps from 1 to 10 MHz. The displacement was measured by applying a 2 *V*_pp_ sinusoidal signal at the first resonant frequency of the pMUT. To confirm uniform activation of the pMUT elements, the surface profile of the pMUT array with 42 elements was measured by SLDV by applying a 2 *V*_pp_ sinusoidal signal at the first resonant frequency.

### Acoustic characterization of the pMUT

The transmit performance of the fabricated pMUT was evaluated using a needle-type hydrophone (HNP-0400; Onda Corp.) with an acoustic intensity measurement system (AIMS III; Onda Corp.). The pMUT array mounted on the PCB was aligned horizontally with the hydrophone in the AIMS chamber filled with deionized water. The acoustic pressure for driven voltage was measured at 5 mm by applying 10-–40-*V*_pp_ using a function generator (33500B; Keysight, Santa Rosa, CA, USA) with an RF amplifier (HSA4101; NF Corp., Yokohama, Japan). The peak pressure of the pMUT as a function of horizontal distance obtained using a hydrophone (5–20 mm) was characterized by applying 40-*V*_pp_ at 9 MHz. The position of the hydrophone for measuring the pressure distribution was controlled by a three-axis motor controller included in the AIMS scanning tank. The loss of acoustic pressure by the cell culture dish used for cell stimulation was examined using a needle-type hydrophone after placing the dish on the surface of the pMUT (Fig. 3e).

### SH-SY5Y cell culture

The human neuroblastoma SH-SY5Y cell line was cultured in DMEM/F12 medium (Gibco, Thermo Fisher Scientific, Waltham, MA, USA) supplemented with 10% (v/v) fetal bovine serum (Gibco) and 1% (v/v) penicillin/streptomycin (Gibco). The cells were grown at 37 °C in a humidified incubator under an atmosphere of 5% CO_2_ in the air, and the medium was changed every 2 days. For biocompatibility testing of PLL-SPION clusters in vitro, 1 × 10^5^ cells were seeded in cell culture-treated 96-well flat-bottomed plates. To evaluate the effects of ultrasound stimulation on cell differentiation, 25,000 cells/mL were plated in 35 mm tissue culture-treated cell culture imaging dishes (μ-Dish, ibidi, Fitchburg, WI, USA). Two days after plating, the growth medium was changed to a differentiation medium supplemented with 10 μM RA as a chemically differentiated control group (RA treatment group). The culture medium was changed every 2 days during incubation for 9 days in all experimental groups.

### Ultrasound stimulation of SH-SY5Y cells for differentiation

SH-SY5Y cells were used to confirm acoustic cell differentiation with the experimental setup schematically illustrated in Fig. 4a. The pulsed ultrasound generated by the pMUT was delivered to the cell dish with SH-SY5Y cells at the site of interest from the bottom of the dish. The gap (~1 mm) between the cell culture dish due to the insulation mold for the PCB wire and pMUT was filled with deionized water. For stimulation, pulsed ultrasound was generated by a function generator in burst excitation mode (*f* = 9 MHz, 15-*V*_pp_, 200 μs pulse with repetition frequency of 1 kHz) with four pMUT channels for different exposure times (10, 20, and 40 min) at room temperature. To achieve a sufficient ultrasound stimulation area, the four pMUT channels at the target region were activated by sequential driving pulses. The total pulse width was 800 μs, with no interphase gap.

### Synthesis of SPION clusters

SPION clusters for Cellbot fabrication were synthesized using a hydrothermal method described previously^42^. Initially, 3 mmol of FeCl_3_ ∙ 6H_2_O, 6 mmol of sodium citrate tribasic dihydrate, 9 mmol of urea, and 60 nmol of polyacrylamide were dissolved and mixed in 60 mL of deionized water. The prepared solution was transferred to a polytetrafluoroethylene-lined autoclave and heated at 200 °C for 19 h. Following the reaction, the autoclave was cooled to room temperature. The SPION clusters were then washed several times with deionized water and ethanol using a magnet. The resultant clusters were dispersed in deionized water to a concentration of 10 mg/mL for future use.

### PLL functionalization of SPION clusters

PLL (P9155; Sigma-Aldrich, St. Louis, MO, USA) was used to functionalize the surface of SPION clusters. The prepared SPION cluster solution (0.5 mL, 10 mg/mL) was added to a mixture of 0.675 mL of PLL solution (1 mg/mL) and 0.075 mL of deionized water. For uniform coating of the SPION clusters with PLL, sonication was applied to the mixed solution for 4 h, and then used in cell experiments after resuspension in a cell culture medium with different concentrations of SPION clusters (0 –150 μg/mL).

### Cell viability testing

The viability of SH-SY5Y cells was measured using a live/dead cell viability kit (LIVE/DEAD; Invitrogen, Carlsbad, CA, USA). SH-SY5Y cells were cultured on 96-well plates for 48 h after adding PLL-SPION clusters at various concentrations (25, 50, 75, 100, 125, and 150 μg/mL). A mixed solution of green and red fluorescence probes was added to wells containing equal volumes of cell culture medium and incubated for 30 min at 37 °C, 5% CO_2_, and 95% humidity. Live/dead fluorescence images of stained cells were obtained using an inverted microscope (Lionheart LX; BioTek, Winooski, VT, USA). The cytotoxicity of PLL-SPION clusters as a function of concentration (25, 50, 75, 100, 125, and 150 μg/mL) was evaluated using an ATP assay kit (CellTitor-Glo; Promega, Madison, WI, USA). The ATP luminescence reagent was added to wells with an equal volume of cell culture medium and incubated for 20 min at room temperature. The luminescence of experimental groups was measured using an ultraviolet-visible light spectrometer (Synergy HTX Multi-Mode Reader; BioTek).

### Fabrication of Cellbots

The Cellbots were fabricated as described previously^13^. SH-SY5Y cells were cultured in 100-mm cell culture dishes (Nunclon Delta Surface; Thermo Fisher Scientific). After washing with phosphate-buffered saline (PBS) to remove the residue, 10 mL of cell culture medium containing PLL-SPION clusters (100 μg/mL) was added to the dishes and incubated at 37 °C, 5% CO_2_, and 95% humidity for 4 h. Cell culture medium with PLL-SPION clusters was exchanged for medium without clusters, and SH-SY5Y cells transfected with PLL-SPION clusters were incubated for 20 h. Then, the Cellbots were trypsinized with 0.25% ethylenediaminetetraacetic acid and suspended in the culture medium. The suspended Cellbots were used for magnetic manipulation experiments.

### Magnetic manipulation of cellbots

Cellbots were manipulated in confocal dishes filled with cell culture medium using an electromagnetic actuation (EMA) system (NanoMag; MagnebotiX, Zurich, Switzerland). The eight electromagnetic coils of the EMA system were configured in a hemispherical shape, and a magnetic field was generated by individually adjusting the current in each coil. The velocity of a single Cellbot during rolling motion was evaluated by applying a rotating magnetic field of 10, 15, or 20 mT at 1–30 Hz. For magnetic manipulation to the target region, the Cellbot was driven by applying a rotating magnetic field of 20 mT at 10 Hz.

### Immunostaining

Non-stimulated and stimulated SH-SY5Y cells were stained on day 7 for β-*III tubulin (TUBB3;* Abcam, Cambridge, UK) as a neuronal-specific marker (green) and 4′,6-diamidino-2-phenylindole (DAPI; Thermo Fisher Scientific) for nuclei (blue) after 2 cycles of ultrasound stimulation. Non-stimulated and stimulated SH-SY5Y cells were fixed with 4% paraformaldehyde and washed with PBS after 1 h. The cells were permeabilized with 1% Triton X-100 in PBS and blocked with 4% bovine serum albumin in a blocking buffer consisting of 10% normal goat serum in PBS. Then, the anti-β-III tubulin antibody was diluted to 1:500 in a blocking buffer and added to the cells. After overnight incubation at 4 °C, the cells were washed twice with 0.2% Triton X-100 in PBS and once with a blocking buffer. The cells were then stained with an anti-rabbit antibody (Alexa Fluor 488; Thermo Fisher Scientific) diluted to 1:500 in a blocking buffer. After washing twice with 0.2% Triton X-100 in PBS, DAPI solution (1:1,000 in PBS with 0.2% Triton X-100) was added. The cells were washed with PBS after 1 h. Fluorescence images of the stained cells were obtained using a confocal microscope (LSM 780; Carl Zeiss, Oberkochen, Germany). The neuronal morphology and neurite length were measured on the images to determine the effects of ultrasound stimulation. Three images collected from three cell culture dishes for each experimental group (non-stimulated, stimulated, and RA treatment) for a total of nine images per condition were evaluated to determine neurite length.

## Supplementary information

Video

SUPPLEMENTAL MATERIAL
