# Supplementary material for: Localized ultrasonic stimulation using a piezoelectric micromachined ultrasound transducer array for selective neural differentiation of magnetic cell-based robots
Source: Microsyst Nanoeng. 2025 Mar 20;11:52. doi: 10.1038/s41378-025-00900-y (PMC11926166; doi:10.1038/s41378-025-00900-y)
Supplement: Supplementary file 2 — SUPPLEMENTAL MATERIAL [file 41378_2025_900_MOESM2_ESM.docx]

**Supplemental information**

Localized ultrasonic stimulation using a piezoelectric micromachined ultrasound transducer array for selective neural differentiation of magnetic cell-based robots

Seonhyoung Kim^1^, Dong-in Kim^1^, Hong Goo Yeo^2^, Gyudong Lee^3^, Jin-young Kim^4^, and Hongsoo Choi^1,5^

^1^ Department of Robotics & Mechatronics Engineering, Daegu Gyeongbuk Institute Science and Technology (DGIST), Daegu 42988, Republic of Korea

^2^ Department of Advanced Materials Engineering, Sun Moon University, Asan-si 31460, Republic of Korea

^3^ Division of Nanotechnology, Daegu Gyeongbuk Institute Science and Technology (DGIST), Republic of Korea

^4^ Division of Biotechnology, Daegu Gyeongbuk Institute Science and Technology (DGIST), Republic of Korea

^5^ DGIST-ETH Microrobotics Research Center, Daegu 42988, Republic of Korea

Correspondence: Hongsoo Choi (mems@dgist.ac.kr)

**Supplementary Fig. 1.** Optical images of SH-SY5Y cells after ultrasound stimulation as a function of input voltage for 10 min. **a** Optical image after ultrasound stimulation with 10 V. **b** Optical image after ultrasound stimulation with 15 V. **c** Optical image after ultrasound stimulation with 20 V.

**
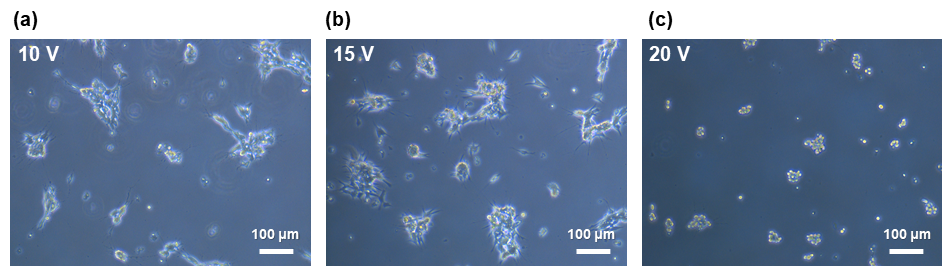
**

**Supplementary Fig. 2.** Evaluation of the effects of ultrasound stimulation using the pMUT array with SH-SY5Y cells. **a** Immunofluorescence images of SH-SY5Y cells labeled with anti-β-III tubulin antibody (green) and stained with DAPI (blue) in non-stimulated (Ctrl.), ultrasound stimulated with continuous waveform (CW) for 10 min, pulsed waveform for 10 min (p10) and 20 min (p20), and retinoic acid (RA) treatment groups. b Mean neurite lengths of SH-SY5Y cells determined from immunofluorescence images (n = 3 dishes in all comparisons). ** P < 0.01; **** P < 0.0001.

**
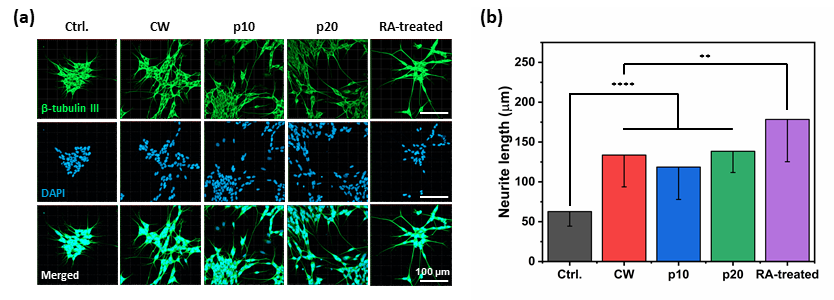
**

**Supplementary Fig. 3.** TEM images of PLL clusters before and after PLL functionalization. **a** Bare SPION clusters. **b** PLL-functionalized SPION clusters.


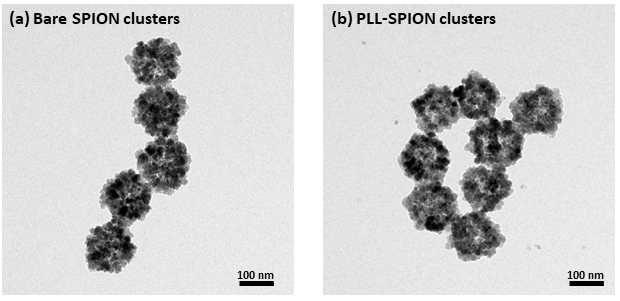


**Supplementary Fig. 4.** Characterziation of hydrodynamic size and Zeta potential after PLL functionalization. **a** dynamic light scattering (DLS) measurements of SPION clusters before and after PLL functionalization. **b** Zeta-potential measurements of SPION clusters before and after PLL functionalization.


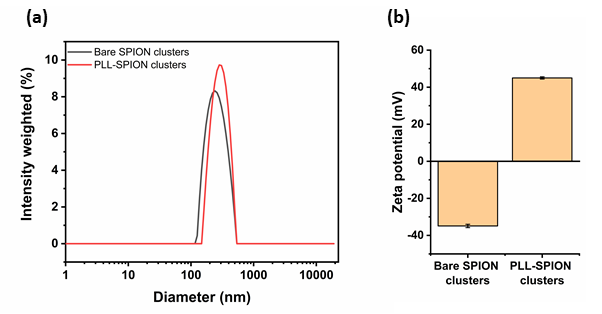


**Supplementary Fig. 5.** Optical images of primary hippocampal neurons before and after ultrasound stimulation **a** Optical image of hippocampal neurons before ultrasound stimulation. **b** Optical image of hippocampal neurons after ultrasound stimulation.

**
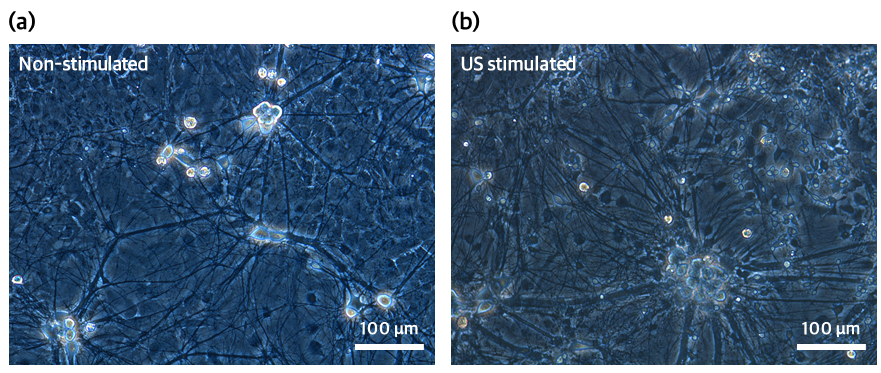
**

**Supplementary Fig. 6.** Schematic illustration of selective ultrasonic cell differentiation using an integrated system with pMUT-based miniaturized stimulation and an electromagnetic actuation system in vivo.


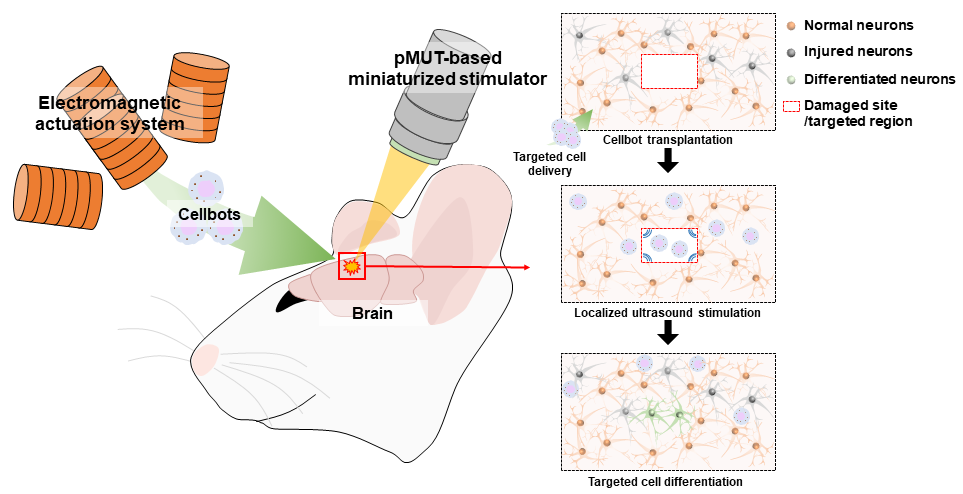


***Supplementary Fig. 7.*** *Acoustic beam directivity of 4 pMUT channels with 20 Vpp at 9 MHz.* ***a*** *Acoustic beam directivity in XY plane.* ***b*** *Acoustic beam directivity in YZ plane.*
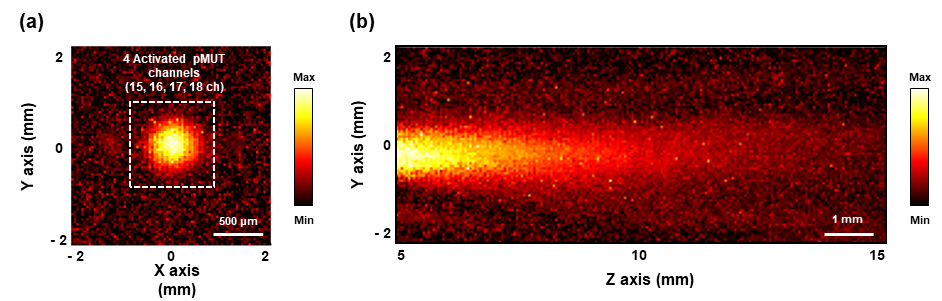


**Supplementary Video 1.**

Magnetic manipulation of Cellbots using a rotating magnetic field
